# Analysis of Resistance Gene Diversity in the Intestinal Microbiome of Broilers from Two Types of Broiler Farms in Hebei Province, China

**DOI:** 10.3390/antibiotics12121664

**Published:** 2023-11-27

**Authors:** Chuncai Liang, Yujie Wei, Xiaolan Wang, Jinduo Gao, Huan Cui, Cheng Zhang, Juxiang Liu

**Affiliations:** College of Veterinary Medicine, Hebei Agricultural University, Baoding 071000, China18333304198@163.com (J.G.);

**Keywords:** broiler, antibiotic resistance genes, mobile genetic elements, high-throughput real-time PCR

## Abstract

The crucial reservoir of antibiotic resistance genes (ARGs) within the chicken intestinal microbiome poses a serious threat to both animal and human health. In China, the overuse of antibiotics has significantly contributed to the proliferation of ARGs in the chicken intestinal microbiome, which is a serious concern. However, there has been relatively little research on the diversity of resistance genes in the chicken intestinal microbiome since the implementation of the National Pilot Work Program for Action to Reduce the Use of Veterinary Antimicrobial Drugs in China. The objective of this study was to analyze the diversity of antibiotic resistance genes carried by the chicken intestinal microbiome in both standard farms (SFs), which implement antibiotic reduction and passed national acceptance, and nonstandard farms (NSFs), which do not implement antibiotic reductions, in Hebei Province. Fresh fecal samples of broiler chickens were collected from SFs (*n* = 4) and NSF (*n* = 1) and analyzed using high-throughput qPCR technology. Our findings revealed that all five farms exhibited a wide range of highly abundant ARGs, with a total of 201 ARGs and 7 MGEs detected in all fecal samples. The dominant ARGs identified conferred resistance to aminoglycosides, macrolide-lincosamide-streptomycin B (MLSB), and tetracycline antibiotics. Cellular protection mechanisms were found to be the primary resistance mechanism for these ARGs. The analysis of the co-occurrence network demonstrated a significant positive correlation between the abundance of MGEs and ARGs. The SF samples showed a significantly lower relative abundance of certain ARGs than the NSF samples (*p* < 0.05). The results of this study show that the abundance of ARGs demonstrated a downward trend after the implementation of the National Pilot Work Program for Action to Reduce the Usage of Veterinary Antimicrobial Drugs in Hebei Province, China.

## 1. Introduction

The emergence of antimicrobial resistance (AMR) poses a grave threat to global public health and has sparked significant concerns regarding the misuse of antibiotics [1]. The World Health Organization calls for the reasonable and standardized usage of medically important antibiotics [2], while several member states, such as European countries and the United States, have also formulated strategies and action plans to combat AMR [3,4].

Antimicrobial drugs are widely used in the livestock breeding industry for disease prevention, treatment, and growth promotion [5,6]. For example, the feed additives tetracyclines (tetracycline, chlortetracycline, oxytetracycline) are commonly administered as antibiotic growth promoters to enhance poultry growth. However, the European Commission has prohibited the use of antibiotics as feed additives since 2006 [7]. Currently, China holds the position of being the leading producer and consumer of antimicrobial drugs worldwide [8]. In China, the use of antibiotics was approximately 162,000 tons in 2013, and more than half of all antibiotics used annually can be attributed to livestock husbandry. This resulted in a further exacerbation of the problem of bacterial resistance in livestock husbandry in China. Therefore, without stringent and effective control measures, it is projected that the use of antibiotics in this industry will increase by 143% from 2010 to 2030 [9]. Moreover, the use of antibiotics in China’s livestock and poultry breeding industry ranks among the highest globally, leaving significant room for reduction efforts [10]. Therefore, China has taken steps to regulate the use of antimicrobial drugs in livestock husbandry; for example, the country issued the “National Action Plan to Curb Bacterial Resistance (2016–2020)” in 2016 and released the National Pilot Work Program for Action to Reduce the Use of Veterinary Antimicrobial Drugs in 2018. Furthermore, the program includes standard acceptance procedures for farms that aim to decrease the usage of antibiotics. The “Evaluation Criteria and Methods for Evaluating the Effectiveness of Reducing the Use of Veterinary Antimicrobial Drugs on Livestock and Poultry Farms” were established to assess the effectiveness of this reduction. These practices aim to guide farms toward rational antimicrobial drug usage while effectively monitoring bacterial resistance, with the ultimate goal of curbing bacterial resistance issues.

The excessive use of antimicrobial drugs has resulted in the perilous emergence of AMR and a significant abundance of ARGs in animal waste [11]. Extensive research has proven that bacterial microorganisms, particularly those found in the intestinal microbiome, are responsible for the dramatic increase in ARGs that go right out into the environment via animal waste and have become a serious worldwide public health problem, posing a significant threat to people’s health [12,13]. ARGs and MGEs are intricately associated with the emergence of AMR. Furthermore, ARGs are transferred between bacteria of the same or different species via MGEs, worsening the spread of ARGs. It is disconcerting to note that livestock and poultry farming employs a greater quantity of antibiotics than that employed in clinical settings for humans. The diverse array of ARGs found within the animal intestinal microbiome suggests an increased susceptibility for horizontal transfer through MGEs. Consequently, substantial attention has recently been directed toward investigating ARGs within the intestinal microbiome [14,15,16].

With the advancement of high-throughput sequencing technology, its adoption has become common in exploring the intestinal microbiome for rapid and comprehensive monitoring of the diversity and abundance of ARGs [17,18,19]. Previous studies have found a necessary connection between the administration of antimicrobial drugs and the abundance of ARGs in the animal intestinal microbiome [20]. After implementing a pilot program aimed at reducing the use of veterinary antimicrobials in SFs, fresh fecal samples of broiler chickens were collected from four SFs and one NSF to assess the effect of the diversity and abundance of ARGs in the broiler intestinal microbiome. Furthermore, high-throughput qPCR technology was employed to characterize the diversity and relative abundance of ARGs and MGEs in the intestinal microbiome of broiler chickens, providing valuable insights into potential implications arising from variations in ARGs and MGEs between these two types of broiler farms, which presents an opportunity for further research and provides a scientific basis for responsible antibiotic use on broiler chicken farms.

## 2. Results

### 2.1. The Diversity of ARGs and MGEs

**Figure 1:**
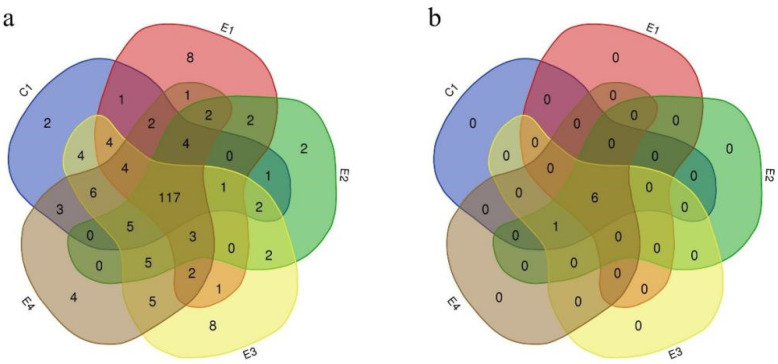
The number of ARGs and MGEs of the five broiler farms that share a relationship. (**a**) ARGs with shared relationships among the five broiler farms. (**b**) MGEs with shared relationships among the five broiler farms. Each circle represents a broiler farm; the circles represent the five broiler farms, with the number inside the overlap indicating shared genes and the numbers outside indicating the unique genes for each farm.

In total, 201 ARGs and 7 MGEs were detected, 117 ARGs (Figure 1a) and 6 MGEs (Figure 1b) of which were found in all fecal samples of the five broiler farms, indicating the diversity of ARGs and MGEs in the broiler intestinal microbiome, as well as similar patterns of resistance in five broiler farms.

**Figure 2:**
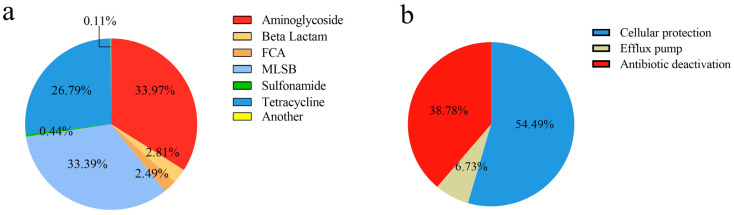
(**a**) The portion of ARGs in the chicken intestinal microbiome of the five broiler farms. (**b**) The portion of the resistance mechanism.

**Figure 3:**
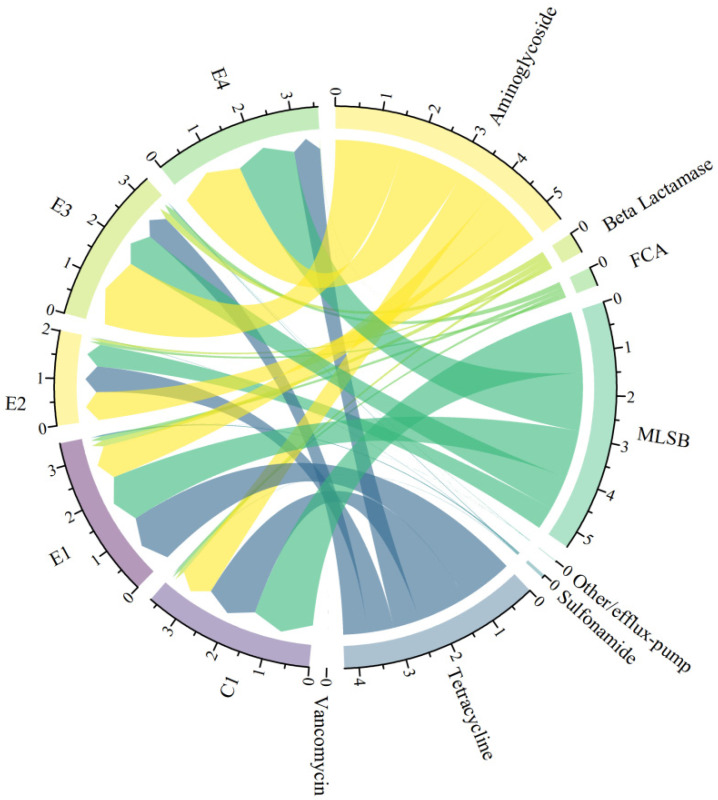
Chord diagram of ARGs in the chicken intestinal microbiome of each broiler farm. Sample information is on the left, and ARG information is on the right; the scale represents the abundance of ARGs in each broiler farm.

All detected ARGs confer resistance to the six major antibiotic classes (Figure 2a), including aminoglycosides, beta-lactams, MLSB (macrolide-lincosamide-streptomycin B), FCA (fluoroquinolone, quinolone, florfenicol, chloramphenicol, and amphenicol), sulfonamides, tetracyclines, and vancomycin. Of these, aminoglycosides (33.97%) were the predominant ARG type, followed by MLSB (33.40%), tetracyclines (26.80%), beta-lactams (2.81%), FCA (2.49%), sulfonamides (0.44%), and vancomycin (0.002%). Similarly, the prevalent gene classes conferring resistance were observed for aminoglycoside, MLSB, and tetracycline antibiotics in the five broiler farms (Figure 3). The identified resistance mechanisms associated with ARGs can be categorized into three types (Figure 2b): antibiotic deactivation, efflux pump mechanism, and cellular protection mechanism. Among these mechanisms of resistance, cellular protection was the main resistance mechanism, accounting for 54.49%, followed by antibiotic deactivation at 38.78%, while efflux pump mechanisms contributed only 6.73%.

Furthermore, among all fecal samples, the top 10 ARGs with relatively high relative abundance primarily consisted of MLSB (*ERMB*, *ERMT-01*, *ERMT-02*), aminoglycosides (*AACA/APHD*, *APHA3-01*, *APHA3-02*, *AADA-01-02*), and tetracyclines (*TETM-01*, *TETO-02*, *TETW-01*). Notably, among these 10 ARGs, the resistance genes *ERMB*, *ERMT-01*, *ERMT-02*, *APHA3-02*, and *TETM-01* were highly abundant in the five broiler farms (Table S4).

### 2.2. Changes in the Abundance of ARGs and MGEs between the SFs and the NSF

**Figure 4:**
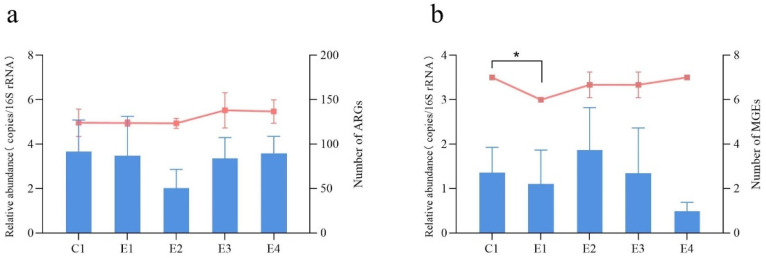
The relative abundance and number of the detected ARGs and MGEs in the five broiler farms. (**a**) The relative abundance and number of the detected ARGs. (**b**) The abundance and number of the detected MGEs. The graph represents the relative abundance; the line graph represents the number. * indicated significant differences (*p* < 0.05) between the broiler farms. C1 represents the NSF, and E1(–)4 represents the SFs.

**Figure 5:**
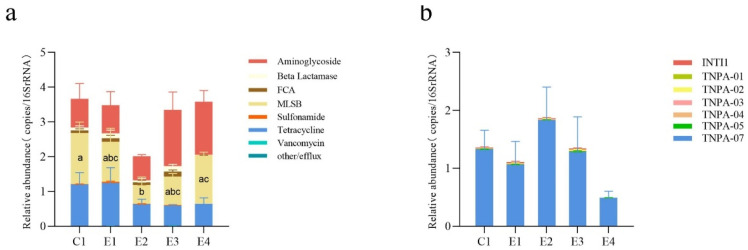
The relative abundance of detected ARGs and MGEs in the five broiler farms. (**a**) The relative abundance of detected ARGs. (**b**) The relative abundance of detected MGEs. The different small letters indicate a significant difference (*p* < 0.05) between the broiler farms. C1 represents the NSF, and E1(–)4 represents the SFs.

The reduction in the use of antibacterial drugs has led to a decrease in the relative abundance of ARGs to some extent. The overall abundance of ARGs detected in E1(–)4 of the SFs was slightly lower than that detected in C1 of the NSF. Among them, the mean abundance of ARGs detected in C1 of the NSF was the highest (3.663 copies/16S rRNA), followed by E4 (3.583 copies/16S rRNA), E1 (3.479 copies/16S rRNA), and E3 (3.350 copies/16S rRNA) of the SFs, while the mean abundance of ARGs in E2 of the SFs was the lowest (2.014 copies/16S rRNA). The average number of detected ARGs in C1, E1, E2, E3, and E4 was 124, 123.7, 123.3, 138, and 136.7, respectively. However, there were no significant differences between the five broiler farms (Figure 4a). At the individual level for specific ARGs, there were significant differences observed among the five broiler farms with regard to their relative abundances. The relative abundance of MLSB resistance genes in E2 of the SFs was considerably lower than that found in both E4 (*p* < 0.05, *p* = 0.038) and C1 (*p* < 0.05, *p* = 0.027) (Figure 5a). Furthermore, the relative abundance of the MLSB resistance gene *ERMB* in both E2 and E3 was significantly reduced compared with those found in C1 (*p* < 0.05) (Table S4).

For the detected MGEs in all fecal samples, *TNPA-07* exhibited the highest relative abundance (97.23%), followed by *INTI1* (1.20%) and *TNPA-02* (0.63%) (Figure S1). Similarly, *TNPA-07* was found to be the most prevalent among five broiler farms (Figure 5b). The mean abundances of MGEs in C1, E1, E2, E3, and E4 were 1.360, 1.106, 1.864, 1.323, and 0.493 copies/16S rRNA, respectively. Furthermore, the average numbers of detected MGEs in C1, E1, E2, E3, and E4 were 7, 6, 6.7, 6.7, and 7, respectively. However, in terms of both the relative abundance and number of detected MGEs, no significant difference was observed between the SFs (E1, E2, E3, E4) and the NSF (C1) (Figure 4b).

The heatmap visually depicts the diversity of ARGs and MGEs across all fecal samples from the five broiler farms (Figure S2). At the level of individual ARGs, different ARGs exhibited distinct responses in terms of relative abundance changes in the five broiler farms. Compared with NSF, SFs showed lower relative abundance for certain specific genes. Different types of broiler farms have significant impacts on the abundance and diversity of ARGs and MGEs, and variations were also observed within sites belonging to the same type of broiler farm. Despite an overall decreasing trend in total ARG abundance, individual ARGs displayed diverse behaviors across the five broiler farms, with some showing substantial decreases while others remained persistent.

The relative abundance and the number of ARGs and MGEs were found to be unrelated. For instance, despite having the highest relative abundance of ARGs, C1 had the lowest number of detected ARGs. In addition, while the relative abundance of MGEs in E2 was higher than that in C1, there was a discrepancy in their detected numbers, with a lower count observed in E2 compared with C1.

### 2.3. Co-occurrence of ARGs and MGEs

**Figure 6:**
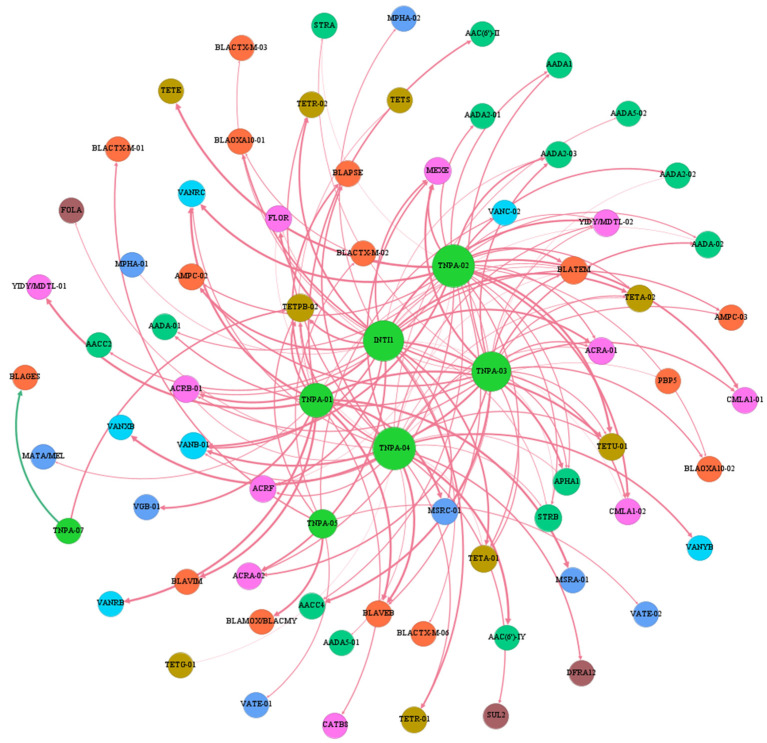
The co-occurrence network of ARGs and MGEs. The node size represents the node degree, and a large size indicates a high degree. The red lines represent a positive correlation, and the green lines represent a negative correlation.

Horizontal gene transfer (HGT) mediated by MGEs plays a crucial role in the transmission of ARGs and facilitates their movement to recipient hosts, leading to the emergence of new antibiotic-resistant bacteria [21]. The co-occurrence network of ARGs and MGEs was visualized based on Spearman correlation analysis to characterize the relationship between the identified ARGs and MGEs. The resulting co-occurrence network consisted of 74 nodes and 137 edges (Figure 6). In this network, *TNPA-02* and *TNPA-04* were identified as key hubs. Among the seven MGEs analyzed, *INTI1*, *TNPA-01*, *TNPA-02*, *TNPA-03*, *TNPA-04*, and *TNPA-05* all exhibited a significant positive correlation with ARGs (*r* > 0.80, *p* < 0.01), particularly gene classes conferring resistance to aminoglycosides and β-lactams. On the other hand, *TNPA-07* showed a significant negative correlation with β-lactam resistance (*BLAGES*) and a positive correlation with the tetracycline resistance gene (*TETPB-01*) but no obvious correlation with other ARGs. Furthermore, we observed that the higher abundance of MGEs was considerably linked with the detected ARG levels. These findings suggest that the increased abundance and diversity of MGEs may further enhance the dissemination and enrichment of ARGs within the chicken intestinal microbiome.

## 3. Materials and Methods

### 3.1. Sample Source

**Figure 7:**
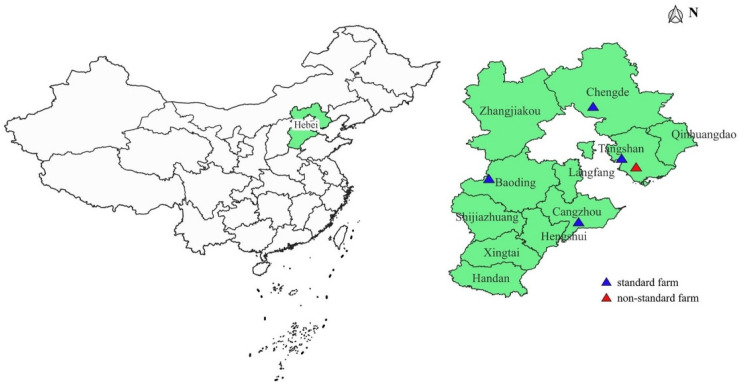
The geographical location of the five broiler farms in the present study.

In total, 15 fresh fecal samples were collected from five broiler farms in the Baoding (114.684007° E, 39.431477° N), Chengde (117.517391° E, 41.059622° N), Tangshan (117.934846° E, 40.185948° N; 118.664675° E, 39.52271° N), and Cangzhou (117.348791° E, 38.157693° N) regions in Hebei Province, China; SFs were labeled E1(–)4, rated as standard farm in 2020, 2019, 2020, and 2021, and one NSF was labeled C1. The locations of the broiler chicken farms are shown in Figure 7, and the basic messages of the five broiler farms are shown in Table S1. At least 5 fresh fecal samples were collected randomly and then mixed into one composite sample. Three composite fecal samples were collected randomly from each broiler chicken farm. The 15 composite fecal samples were promptly stored at − 80 °C for DNA extraction.

### 3.2. DNA Extraction and The Real-Time qPCR

According to the manufacturer’s instructions, DNA was extracted from fecal samples weighing 0.2 g into a 2 mL centrifuge tube using the TIANAMP Stool DNA Kit. The quantity and quality of DNA were determined using a Quawell Q3000 UV spectrophotometer. The qPCR analysis was carried out using a StepOnePlus™ real-time fluorescence quantitative PCR system (Thermo Scientific, Waltham, MA, USA). The qPCR reaction system (10 μL) totaled 5 μL of TB Green Premix Ex Taq II (Tli RNaseH Plus) (2×), 0.4 μL of (10 μM) each upstream and downstream primer, 0.2 μL (50×) ROX Reference Dye, 1 μL of DNA template and 3 µL of ddH_2_O. The qPCR reaction conditions were the following: 95 °C predenaturation was implemented for 10 min, followed by 40 cycles of 95 °C denaturation for 30 s, 60 °C annealing for 30 s, 72 °C extension for 30 s, and 72 °C final extension for 30 s. This study used 271 primer sets targeting 6 major classes of ARGs (aminoglycosides, beta-lactams, MLSB (macrolide-lincosamide-streptomycin B), FCA (fluoroquinolone, quinolone, florfenicol, chloramphenicol, and amphenicol), sulfonamides, tetracyclines, and vancomycin), one transposon gene (*INTI1*), six integron genes (*TNPA-01*, *TNPA-02*, *TNPA-03*, *TNPA-04*, *TNPA-05*, *TNPA-07*), and one *16S rRNA* gene (Table S3). The relative abundance and fold change of ARGs in fecal samples were calculated based on the following method:

ΔCt = Ct (ARG) − Ct (*16S rRNA*), (1)

ΔΔCt = ΔCt (treatment) − Ct (control),(2)

FC = 2^−ΔΔCt^, (3)

Relative abundance = 2^−ΔCt^, (4)

The threshold cycle (Ct) of 40 was the detection limit. ARGs were the target genes, and *16S rRNA* was the internal reference gene [22]. The treatment group comprises E1(–)4 of the SFs, and the control group includes C1 of the NSF.

### 3.3. Statistical Analysis

The difference in the abundance of ARGs and MGEs in the five broiler farms was analyzed using Student’s *t* test performed with SPSS 25.0. The heatmap of ARGs and MGEs in the five broiler farms was analyzed using the heatmap package of R software (3.6.3). Pearson analysis was applied to characterize the relationship between the abundance of ARGs and MGEs. The co-occurrence network of ARGs and MGEs was based on Pearson analysis and constructed using Gephi software (0.10.1).

## 4. Discussion

China, being the largest consumer of chicken products globally, faces a significant challenge to human health due to the improper utilization and overuse of antimicrobial drugs in chicken farms. To address this issue, our study employed high-throughput qPCR technology to characterize the diversity and relative abundance of ARGs and MGEs within the broiler chicken intestinal microbiome from two distinct types of broiler chicken farms located in Hebei Province, China.

Antibiotics are commonly used as growth promoters in the chicken breeding industry to stimulate growth. In addition to the excessive administration of coccidiostats, macrolides, penicillin, and tetracyclines in this industry has a significant impact on antimicrobial resistance and public health [23]. Among small- and medium-sized farms in the chicken breeding industry, amoxicillin of β-lactams is the most frequently used antibiotic, followed by the fluoroquinolone antibiotics norfloxacin and ofloxacin [24]. The widespread use of antibiotics is primarily responsible for the emergence of resistance genes. As a result of continuous large-scale antibiotic usage in poultry farming, not only have residues increased but the abundance of ARGs in chickens has also risen significantly, posing a serious threat to human health [25]. In line with previous research findings, our study detected various ARGs conferring resistance to MLSB (macrolide-lincosamide-streptogramin B), aminoglycosides, and tetracyclines within the chicken intestinal microbiome across five broiler farms. Tong et al. [26] found that ARGs conferring resistance to cephalosporins, aminoglycosides, and tetracyclines were the most prevalent in the chicken intestinal microbiome due to selective pressure exerted by antibacterial drugs. Other studies have also identified tetracycline, aminoglycoside, and MLSB resistance genes as major ARGs within the chicken intestinal microbiome [27,28]. Moreover, aminoglycosides, MLSB, and tetracyclines are the predominant resistance genes found in animal feces, as well as in the surrounding environment, including water, soil, and air. Similarly, poultry, cattle, and swine fecal samples frequently exhibit ARGs conferring resistance to aminoglycoside (42%), MLSB (19%), tetracyclines (15%), and multiple drugs (14%) [29,30]. Previous studies have indicated that sulfonamide, macrolide, tetracycline, β-lactam, and aminoglycoside resistance genes are highly prevalent in wastewater from livestock farms [31,32]. Moreover, the abundance of tetracycline resistance genes was significantly higher in samples collected closest to feed yard boundaries compared with particulate matter samples gathered at other distances [33]; this finding is consistent with previous reports on soil samples, where tetracycline resistance genes were commonly detected [34,35]. Therefore, it is imperative to minimize the use of aminoglycosides, MLSB, and tetracycline antibiotics in broiler breeding practices. Additionally, proactive measures should be taken to prevent disease outbreaks promptly and select suitable antibiotic alternatives. This approach is crucial for averting the exacerbation of bacterial resistance and the dissemination of resistance genes.

The study conducted found that all fecal samples collected from the different types of broiler farms (four SFs and one NSF) located in Hebei Province, China, exhibited a substantially high relative abundance of detected ARGs in the broiler intestinal microbiome. This finding clearly indicates that ARGs continue to be widespread, and antibiotic resistance remains a critical concern in broiler farms. This observation can be attributed to the long-term administration of antimicrobial drugs in poultry farming over the course of 50 years, leading to the high level of resistance genes in the chicken intestinal microbiome, regardless of antibiotic pressure [36,37]. Moreover, it is worth noting that some environmental bacteria inherently carry ARGs [38]. This result is similar to other studies’ findings in that ARGs were still detected even without antibiotic pressure [28,39]. The abundance of ARGs is influenced by two categories of factors. Direct factors involve selection pressure on the ARGs themselves, while indirect factors involve selection pressure that affects the microbial composition and subsequently influences the abundance of ARGs [40,41], such as feeding practices, diets, and farm environment-associated ARGs [42,43]. Several studies have shown that the presence of heavy metal residues in soil, water, and feces can further contribute to the proliferation and enrichment of drug resistance genes. Unlike antibiotics, heavy metals, such as copper (Cu) and zinc (Zn), are frequently used as feed additives in animal husbandry. These heavy metals cannot be degraded in the environment; this means that they can exert continuous pressure on antibiotic resistance genes and increase the abundance of ARGs through co-selection and cross-selection. Moreover, heavy metals such as Cu were found to have a significant positive correlation with transposons, which suggests that the presence of heavy metal residues may promote the HGT of ARGs in bacterial communities [44].

The transfer of ARGs between bacteria through MGEs can give rise to the emergence of multidrug-resistant pathogenic bacteria, posing a serious threat to human health and ecological security [35,45]. In the analysis of the abundance and diversity of MGEs, a high relative abundance of MGEs was observed in all five broiler farms; furthermore, a positive correlation between MGEs and ARGs was noted, potentially leading to the dissemination and enrichment of ARGs within the chicken intestinal microbiome. In line with previous research findings, the migration and spread of ARGs have been closely associated with MGEs [46]. Moreover, some research suggests that there may be potential co-occurrence patterns between MGE and ARG subtypes; this means that they can spread together and can lead to the enrichment of multiple ARGs when one type of antibiotic is present [29]. Similar results also indicated that integrons have significant positive correlations with many ARGs and can also often coexist with ARGs, such as integron *int1* and ARGs (*ermC*, *qnrA*) [47]. Similarly, transposons, as typical MGEs, play a significant role in the HGT of ARGs and co-occur with ARGs (*aadA*, *tetH*, *tetM*) through *tnpA* [48].

Previous research has demonstrated that the main consequence of antibiotic misuse is the creation of induced ARGs. By reducing the use of antibiotics, we can effectively mitigate the spread of antibiotic resistance [49]. The abundance of ARGs may be influenced by the duration of antimicrobial reduction implementation. In this study, it is evident that there is a correlation between the relative abundance of resistance genes and the duration of the antimicrobial reduction plan. Among the five broiler farms, ARGs were found to be most prevalent in C1 of the NSF. Additionally, among the four SFs, E4 exhibited the highest relative abundance of ARGs, followed by E1 and E3. However, E2 had the lowest relative abundance among these four SFs. Therefore, it can be inferred that implementing an antimicrobial reduction plan can significantly reduce drug resistance gene epidemics to some extent. Furthermore, longer implementation periods are associated with lower relative abundances of resistance genes in the chicken intestinal microbiome. Consistent with previous research findings, this research assessed the effects of colistin withdrawal and found that colistin resistance and the abundance of the *mcr-1* gene showed a significant decreasing trend in *Escherichia coli* from animals and humans between 2015 and 2019 [50]. Similar results also indicated that a ban on the use of colistin in animal feed could effectively contribute to reducing the prevalence of the *mcr-1* gene [51]. What’s more, the trend of antimicrobial resistance has decreased since the use of antibiotics as feed additives was prohibited in China in 2020, although no fundamental change has occurred [15].

At the level of individual ARGs, certain ARGs exhibited similar responses to the overall abundance changes in resistance genes across the five broiler farms, and the abundance of specific ARGs was significantly lower in SFs compared with NSF. Statistical analyses indicated that the relative abundance of MLSB resistance genes was significantly reduced after plan implementation, but MLSB resistance genes were still prevalent in the chicken intestinal microbiome of the five broiler farms. This finding shows that, after implementing the antibiotic reduction plan, there may have been a decrease in the use of MLSB antibiotics in the SFs compared with the NSF. The reduction in antibiotic usage not only impacts the overall levels of ARGs but also primarily reduces the relative abundance of specific ARG subtypes [52]. In this study, a downward trend was observed in the relative abundance of ARGs in the SFs after the implementation of the antimicrobial reduction plan, but the difference was not obvious. This could be attributed to the short period (3–4 years) of the implementation of antimicrobial reduction and regional variations in medication habits in different regions. However, due to the short duration of antimicrobial reduction implementation, it remains uncertain whether the implementation had a significant impact on the diversity and abundance of ARGs in the chicken intestinal microbiome. Therefore, further monitoring and research are necessary to gain a comprehensive understanding of the effects.

It is important to note that there are some limitations to our study. Firstly, the analysis was carried out on only one NSF, as most large-scale farms have already implemented antimicrobial reduction. Secondly, only 15 fecal samples were analyzed. We aim to broaden the sample range and increase the number of NSFs in future long-term studies so as to make the conclusions of our study broader and more reliable.

## 5. Conclusions

In this study, we investigated and analyzed the diversity and abundance of the detected ARGs in the chicken intestinal microbiome from two types of broiler farms in Hebei Province. Our findings indicate that the implementation of antimicrobial reduction strategies can effectively decrease the abundance of ARGs in the chicken intestinal microbiome, thereby mitigating the spread of antibiotic resistance, particularly for MLSB resistance genes. It is imperative to conduct future studies with a larger sample size to explore the long-term effects of antimicrobial reduction.

## Data Availability

The study’s original contributions are included in the article; further inquiries can be directed to the corresponding authors.

## Supplementary Materials

The following supporting information can be downloaded at: https://www.mdpi.com/article/10.3390/antibiotics12121664/s1, Figure S1: The composition of ARGs in the chicken intestinal microbiome in five broiler farms; Figure S2: The heatmap of ARGs and MGEs in five broiler farms; Table S1: Characteristics of the six broiler chicken farms in Hebei Province, China; Table S2: Information on antibiotic treatments in the 5 sampling farms during the study period; Table S3: Primers for antibiotic resistance genes, mobile genetic elements and 16S rRNA gene sequence used in this study; Table S4: The relative abundance of antibiotic resistance genes and mobile genetic elements.
