# Supplementary material for: Analysis of Resistance Gene Diversity in the Intestinal Microbiome of Broilers from Two Types of Broiler Farms in Hebei Province, China
Source: Antibiotics (Basel). 2023 Nov 27;12(12):1664. doi: 10.3390/antibiotics12121664 (PMC10741226; doi:10.3390/antibiotics12121664)
Supplement: Supplementary file 1 [file antibiotics-12-01664-s001.zip › antibiotics-2729792-supplementary.pdf]

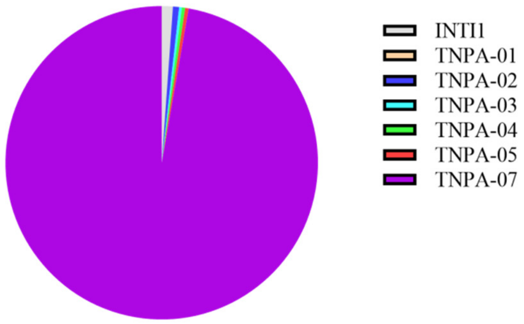

Figure S1. The composition of ARGs in the chicken intestinal microbiome in five broiler farms.

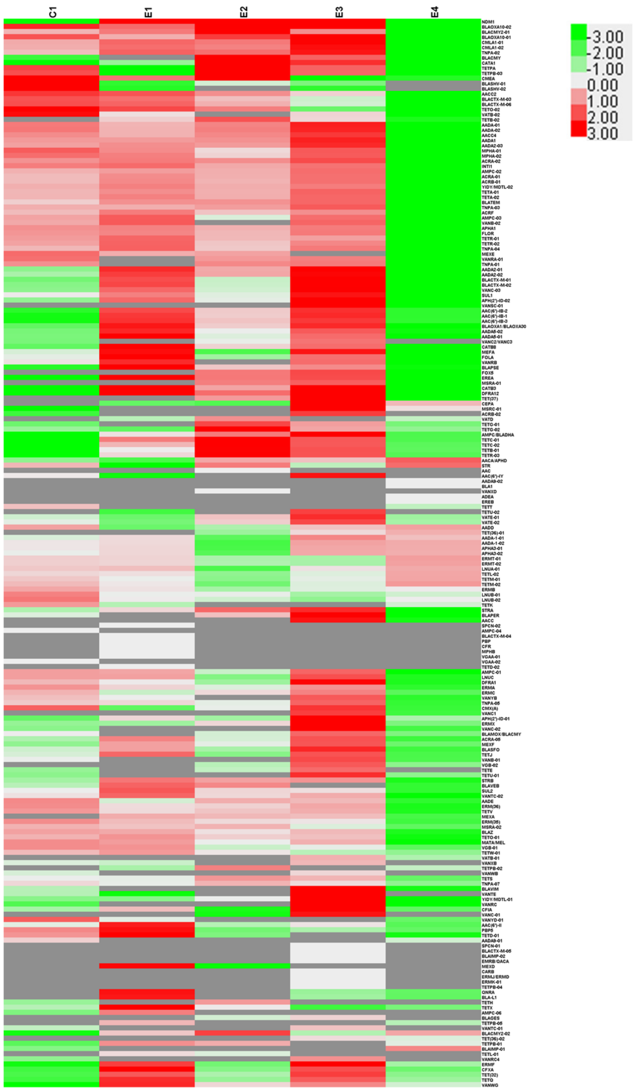

Figure S2. The heatmap of ARGs and MGEs in five broiler farms.

Table S1. Characteristics of the six broiler chicken farms in Hebei Province, China.

	E1	E2	E3	E4	C1
Type	SF	SF	SF	SF	NSF
geographical location	Tangshan	Chengde	Cangzhou	Baoding	Tangshan
Latitude and longitude	117.934846°E, 40.185948°N	117.517391°E, 41.059622°N	117.348791°E, 38.157693°N	114.684007°E, 39.431477°N	118.664675°E, 39.52271°N
Time to be standard (Unit: year)	2020	2019	2020	2021	-
Number of barn	12	6	8	9	10
Fattening Scale per barn	1600 ㎡	1500 ㎡	1440 ㎡	1600 ㎡	1600 ㎡
Animal quantity per barn (Unit: thousand)	30	40	30	30	33
Chicken breed	Arbor Acres broiler	Arbor Acres broiler	Arbor Acres broiler	Arbor Acres broiler	Cobb broiler
The age of chicken (Unit:Day)	39	39	39	39	39
Animal habitat	Cages	Cages	Cages	Cages	Cages
Ventilation	Draught fans	Draught fans	Draught fans	Draught fans	Draught fans
Feeding patterns	Automatic	Automatic	Automatic	Automatic	Automatic

Note: The effectiveness of reducing antibiotic use in broiler farms is evaluated based on four aspects: the farm's basic conditions, regulations, relevant records, and the efficacy of reduction actions. The farm is inspected, scored, and evaluated, if the score is 80 or higher, it is considered a "Standard Farm" (SF). Not implemented antimicrobial reduction, it is considered a "Non-Standard Farm" (NSF). The basic requirements for the use of antimicrobial agents in SFs are as follows: the amount of antimicrobial agents used per ton of broiler (gross weight) should be limited to 100g over a growth period of no more than 60 days.

Table S2. Information on antibiotic treatments in the 5 sampling farms during the study period.

	E1	E 2	E 3	E 4	C1
1-5	Lincomycin	Florfenicol	Cefalexin	Ampicillin	Cefalexin
7-10	-	-	-	-	-
12-15	-	-	-	-	Ceftriaxone, Levofloxacin
18-21	Sulfachloropyrazine sodium, Neomycine	Apramycine	-	Florfenicol	-
26-29	-	-	Levofloxacin, Ceftriaxone	-	-
32-35	Lincomycin, Doxycycline	-	-	-	Lincomycin, Amikacin

Table S3. Primers for antibiotic resistance genes, mobile genetic elements and 16S rRNA gene sequence used in this study.

Gene name	Primer	Classification
16s rRNA	F: GGGTTGCGCTCGTTGC<br>R: ATGGYTGTCGTCAGCTCGTG	
aac	F: CCCTGCGTTGTGGCTATGT<br>R: TTGGCCACGCCAATCC	Aminoglycoside
aac(6')-I1	F: GACCGGATTAAGGCCGATG<br>R: CTTGCCTTGATATTCAGTTTTTATAACCA	Aminoglycoside
aac(6')-Ib(aka aacA4)-01	F: GTTTGAGAGGCAAGGTACCGTAA<br>R: GAATGCCTGGCGTGTTTGA	Aminoglycoside
aac(6')-Ib(aka aacA4)-02	F: CGTCGCCGAGCAACTTG<br>R: CGGTACCTTGCCTCTCAAACC	Aminoglycoside
aac(6')-Ib(aka aacA4)-03	F: AGAAGCACGCCCGACACTT<br>R: GCTCTCCATTCAGCATTGCA	Aminoglycoside
aac(6')-II	F: CGACCCGACTCCGAACAA<br>R: GCACGAATCCTGCCTTCTCA	Aminoglycoside
aac(6')-IY	F: GCTTTGCGGATGCCTCAAT<br>R: GGAGAACAAAAATACCTTCAAGGAAA	Aminoglycoside
aacA/aphD	F: AGAGCCTTGGGAAGATGAAGTTT	Aminoglycoside

	R: TTGATCCATACCATAGACTATCTCATCA	
aacC	F: CGTCACTTATTCGATGCCCTTAC<br>R: GTCGGGCGCGGCATA	Aminoglycoside
aacC1	F: GGTCGTGAGTTCGGAGACGTA<br>R: GCAAGTTCCCGAGGTAATCG	Aminoglycoside
aacC2	F: ACGGCATTCTCGATTGCTTT<br>R: CCGAGCTTCACGTAAGCATTT	Aminoglycoside
aacC4	F: CGGCGTGGGACACGAT<br>R: AGGGAACCTTTGCCATCAACT	Aminoglycoside
aadA-01	F: GTTGTGCACGACGACATCATT<br>R: GGCTCGAAGATACCTGCAAGAA	Aminoglycoside
aadA-02	F: CGAGATTCTCCGCGCTGTA<br>R: GCTGCCATTCTCCAAATTGC	Aminoglycoside
aadA1	F: AGCTAAGCGCGAACTGCAAT<br>R: TGGCTCGAAGATACCTGCAA	Aminoglycoside
aadA-1-01	F: AAAAGCCCGAAGAGGAACTTG<br>R: CATCTTTCACAAAGATGTTGCTGTCT	Aminoglycoside
aadA2-01	F: ACGGCTCCGCAGTGGAT<br>R: GGCCACAGTAACCAACAAATCA	Aminoglycoside
aadA2-02	F: CTTGTCGTGCATGACGACATC<br>R: TCGAAGATACCCGCAAGAATG	Aminoglycoside
aadA2-03	F: CAATGACATTCTTGCGGGTATC<br>R: GACCTACCAAGGCAACGCTATG	Aminoglycoside
aadA5-01	F: ATCACGATCTTGCGATTTTGCT<br>R: CTGCGGATGGGCCTAGAAG	Aminoglycoside
aadA5-02	F: GTTCTTGCTCTTGCTCGCATT<br>R: GATGCTCGGCAGGCAAAC	Aminoglycoside
aadA9-01	F: CGCGGCAAGCCTATCTTG<br>R: CAAATCAGCGACCGCAGACT	Aminoglycoside
aadA9-02	F: GGATGCACGCTTGGATGAA	Aminoglycosid

	R: CCTCTAGCGGCCGGAGTATT	e
aadD	F: CCGACAACATTTCTACCATCCTT	Aminoglycoside
	R: ACCGAAGCGCTCGTCGTATA	
aadE	F: TACCTTATTGCCCTTGGAAGAGTTA	Aminoglycoside
	R: GGAACTATGTCCCTTTTAATTCTACAATCT	
acrA-01	F: CAACGATCGGACGGGTTTC	FCA
	R: TGGCGATGCCACCGTACT	
acrA-02	F: GGTCTATCACCCTACGCGCTATC	FCA
	R: GCGCGCACGAACATACC	
acrA-03	F: CAGACCCGCATCGCATATT	FCA
	R: CGACAATTTCGCGCTCATG	
acrA-04	F: TACTTTGCGCGCCATCTTC	FCA
	R: CGTGCGCGAACGAACAT	
acrA-05	F: CGTGCGCGAACGAACA	FCA
	R: ACTTTGCGCGCCATCTTC	
acrB-01	F: AGTCGGTGTTCGCCGTTAAC	FCA
	R: CAAGGAAACGAACGCAATACC	
acrB-02	F: TGGTAGTGGGCGTCATTAACAC	FCA
	R: GGCAACGTAATCCGAAATATCC	
acrF	F: GCGGCCAGGCACAAAA	FCA
	R: TACGCTCTTCCCACGGTTTC	
adeA	F: CAGTTCGAGCGCCTATTTCTG	FCA
	R: CGCCCTGACCGACCAAT	
ampC/blaDHA	F: TGGCCGCAGCAGAAAGA	Beta Lactamase
	R: CCGTTTTATGCACCCAGGAA	
ampC-01	F: TGGCGTATCGGGTCAATGT	Beta Lactamase
	R: CTCCACGGGCCAGTTGAG	
ampC-02	F: GCAGCACGCCCCGTAA	Beta Lactamase
	R: TGTACCCATGATGCGCGTACT	
ampC-03	F: AACAAAAGATCCCCGGTATGG	Beta Lactamase
	R: ACGCCCGTAAATGTTTTGCT	
ampC-04	F: TCCGGTGACGCGACAGA	Beta Lactamase
	R: CAGCACGCCGGTGAAAGT	
ampC-05	F: CTGTTCGAGCTGGGTTCTATAAGTAAA	Beta Lactamase
	R: CAGTATCTGGTCACCGGATCGT	
ampC-06	F: CCGCTCAAGCTGGACCATAC	Beta Lactamase
	R: CCATATCCTGCACGTTGGTTT	
ampC-07	F: CCGCCCAGAGCAAGGACTA	Beta Lactamase
	R: GCTCGACTTCACGCCGTAAG	
ampC-08	F: GCAGCGAAGCGTCAGTCA	Beta Lactamase
	R: AGATCCGTGGCCGCATAA	

ampC-09	F: CAGCCGCTGATGAAAAAATATG<br>R: CAGCGAGCCCACTTCGA	Beta Lactamase
aph	F: TTTCAGCAAGTGGATCATGTTAAAAT<br>R: CCAAGCTGTTTCCACTGTTTTTC	Aminoglycoside
aph(2′)-Id-01	F: TGAGCAGTATCATAAGTTGAGTGAAAAG<br>R: GACAGAACAATCAATCTCTATGGAATG	Aminoglycoside
aph(2′)-Id-02	F: TAAGGATATACCGACAGTTTTGGAAA<br>R: TTTAATCCCTCTTCATACCAATCCATA	Aminoglycoside
aph6ia	F: CCCATCCCATGTGTAAGGAAA<br>R: GCCACCGCTTCTGCTGTAC	Aminoglycoside
aphA1(aka kanR)	F: TGAACAAGTCTGGAAAGAAATGCA<br>R: CCTATTAATTTCCCCTCGTCAAAAA	Aminoglycoside
aphA3-01	F: AAAAGCCCGAAGAGGAACTTG<br>R: CATCTTTCACAAAGATGTTGCTGTCT	Aminoglycoside
aphA3-02	F: CGGAATTGAAAAAACTGATCGAA<br>R: ATACCGGCTGTCCGTCATTT	Aminoglycoside
bla1	F: GCAAGTTGAAGCGAAAGAAAAGA<br>R: TACCAGTATCAATCGCATATACACCTAA	Beta Lactamase
bla-ACC-1	F: CACACAGCTGATGGCTTATCTAAAA<br>R: AATAAACGCGATGGGTTCCA	Beta Lactamase
blaCMY	F: CCGCGGCGAAATTAAGC<br>R: GCCACTGTTTGCCTGTCAGTT	Beta Lactamase
blaCMY2-01	F: AAAGCCTCATGGGTGCATAAA<br>R: ATAGCTTTTGTTTGCCAGCATCA	Beta Lactamase
blaCMY2-02	F: GCGAGCAGCCTGAAGCA<br>R: CGGATGGGCTTGTCCTCTT	Beta Lactamase
blaCTX-M-01	F: CACAGTTGGTGACGTGGCTTAA<br>R: CTCCGCTGCCGGTTTTATC	Beta Lactamase
blaCTX-M-02	F: GGAGGCGTGACGGCTTTT<br>R: TTCAGTGCGATCCAGACGAA	Beta Lactamase
blaCTX-M-03	F: GCCGCGGTGCTGAAGA<br>R: ATCGGATTATAGTTAACCAGGTCAGATTT	Beta Lactamase
blaCTX-M-04	F: CGATACCACCACGCCGTTA<br>R: GCATTGCCCAACGTCAGATT	Beta Lactamase
blaCTX-M-05	F: CTTGGCGTTGCGCTGAT<br>R: CGTTCATCGGCACGGTAGA	Beta Lactamase
blaCTX-M-06	F: GCGATAACGTGGCGATGAAT	Beta Lactamase

	R: GTCGAGACGGAACGTTTCGT	
blaGES	F: GCAATGTGCTCAACGTTCAAG	Beta Lactamase
	R: GTGCCTGAGTCAATTCTTTCAAAG	
blaIMP-01	F: AACACGGTTTGGTGGTTCTTGTA	Beta Lactamase
	R: GCGCTCCACAAACCAATTG	
blaIMP-02	F: AAGGCAGCATTTCCTCTCATTTT	Beta Lactamase
	R: GGATAGATCGAGAATTAAGCCACTCT	
bla-L1	F: CACCGGGTTACCAGCTGAAG	Beta Lactamase
	R: GCGAAGCTGCGCTTGTAGTC	
blaMOX/blaCMY	F: CTATGTCAATGTGCCGAAGCA	Beta Lactamase
	R: GGCTTGTCCTCTTTCGAATAGC	
blaOCH	F: GGCGACTTGCGCCGTAT	Beta Lactamase
	R: TTTTCTGCTCGGCCATGAG	
blaOKP	F: GCCGCCATCACCATGAG	Beta Lactamase
	R: GGTGACGTTGTCACCGATCTG	
blaOXA1/blaOXA30	F: CGGATGGTTTGAAGGGTTTATTAT	Beta Lactamase
	R: TCTTGGCTTTTATGCTTGATGTTAA	
blaOXA10-01	F: CGCAATTATCGGCCTAGAAACT	Beta Lactamase
	R: TTGGCTTTCCGTCCCATTT	
blaOXA10-02	F: TCAACAAATCGCCAGAGA	Beta Lactamase
	R: TCCCACACCAGAAAAACCA	
blaOXY	F: CGTTCAGGCGGCAGGTT	Beta Lactamase
	R: GCCGCGATATAAGATTTGAGAATT	
blaPAO	F: CGCCGTACAACCGGTGAT	Beta Lactamase
	R: GAAGTAATGCGGTTCTCCTTTCA	
blaPER	F: TGCTGGTTGCTGTTTTTGTGA	Beta Lactamase
	R: CCTGCGCAATGATAGCTTCAT	
blaPSE	F: TTGTGACCTATTCCCCTGTAATAGAA	Beta Lactamase
	R: TGCGAAGCACGCATCATC	
blaROB	F: GCAAAGGCATGACGATTGC	Beta Lactamase
	R: CGCGCTGTTGTCGCTAAA	
blaSFO	F: CCGCCGCCATCCAGTA	Beta Lactamase
	R: GGGCCGCCAAGATGCT	
blaSHV-01	F: TCCCATGATGAGCACCTTTAAA	Beta Lactamase
	R: TTCGTCACCGGCATCCA	
blaSHV-02	F: CTTTCCCATGATGAGCACCTTT	Beta Lactamase
	R: TCCTGCTGGCGATAGTGGAT	
blaTEM	F: AGCATCTTACGGATGGCATGA	Beta Lactamase
	R: TCCTCCGATCGTTGTCAGAAGT	
blaTLA	F: ACACTTTGCCATTGCTGTTTATGT	Beta Lactamase
	R: TGCAAATTTCGGCAATAATCTTT	
blaVEB	F: CCCGATGCAAAGCGTTATG	Beta Lactamase
	R: GAAAGATTCCCTTTATCTATCTCAGACAA	
blaVIM	F: GCACTTCTCGCGGAGATTG	Beta Lactamase

	R: CGACGGTGATGCGTACGTT	
blaZ	F: GGAGATAAAGTAACAAATCCAGTTAGATATGA<br>R: TGCTTAATTTTCCATTTGCGATAAG	Beta Lactamase
carB	F: GGAGTGAGGCTGACCGTAGAAG<br>R: ATCGGCGAAACGCACAAA	MLSB
catA1	F: GGGTGAGTTTCACCAGTTTTGATT<br>R: CACCTTGTCGCCTTGCGTATA	FCA
catB3	F: GGTCATGCCCGGAATCAAGA<br>R: CGATAGCGTAAGGCTCCACA	FCA
catB8	F: CACTCGACGCCTTCCAAAG<br>R: CCGAGCCTATCCAGACATCATT	other/efflux
cepA	F: AGTTGCGCAGAACAGTCCTCTT<br>R: TCGTATCTTGCCCGTCGATAAT	Beta Lactamase
cfiA	F: GCAGCGTTGCTGGACACA<br>R: GTTCGGGATAAACGTGGTGACT	Beta Lactamase
cfr	F: GCAGGTTGGGAGTCATTTTG<br>R: ACGGTTGGCTAGAGCTTCAC	FCA
cfxA	F: TCATTCCTCGTTCAAGTTTTCAGA<br>R: TGCAGCACCAAGAGGAGATGT	Beta Lactamase
cmeA	F: GCAGCAAAGAAGAAGCACCAA<br>R: AGCAGGGTAAGTAAAACTAAGTGGTAAATCT	FCA
cmlA1-01	F: TAGGAAGCATCGGAACGTTGAT<br>R: CAGACCGAGCACGACTGTTG	FCA
cmlA1-02	F: AGGAAGCATCGGAACGTTGA<br>R: ACAGACCGAGCACGACTGTTG	FCA
cmx(A)	F: GCGATCGCCATCCTCTGT<br>R: TCGACACGGAGCCTTGGT	FCA
cphA-01	F: GCGAGCTGCACAAGCTGAT<br>R: CGGCCCAGTCGCTCTTC	Beta Lactamase
cphA-02	F: GTGCTGATGGCGAGTTTCTG<br>R: GGTGTGGTAGTTGGTGTTGATCAC	Beta Lactamase
dfrA1	F: GGAATGGCCCTGATATTCCA<br>R: AGTCTTGCGTCCAACCAACAG	other/efflux
dfrA12	F: CCTCTACCGAACCGTCACACA<br>R: GCGACAGCGTTGAAACAACTAC	other/efflux
emrB/qacA	F: CTTTTCTCTAACCGTACATTATCTACGATAAA<br>R: AGAACGTAGCGACTGATAAAATGCT	FCA
ereA	F: CCTGTGGTACGGAGAATTCATGT<br>R: ACCGCATTCGCTTTGCTT	MLSB
ereB	F: GCTTTATTTCAGGAGGCGGAAT	other/efflux

	R: TTTTAAATGCCACAGCACAGAATC	
erm(34)	F: GCGCGTTGACGACGATTT	MLSB
	R: TGGTCATACTCGACGGCTAGAAC	
erm(35)	F: TTGAAAACGATGTTGCATTAAGTCA	MLSB
	R: TCTATAATCACAACTAACCACTTGAACGT	
erm(36)	F: GGCGGACCGACTTGCAT	MLSB
	R: TCTGCGTTGACGACGGTTAC	
ermA	F: TTGAGAAGGGATTTGCGAAAAG	MLSB
	R: ATATCCATCTCCACCATTAATAGTAAACC	
ermA/ermTR	F: ACATTTTACCAAGGAACTTGTGGAA	MLSB
	R: GTGGCATGACATAAACCTTCATCA	
ermB	F: TAAAGGGCATTTAACGACGAAA	MLSB
	R: TTTATACCTCTGTTTGTTAGGGAATTGAA	
ermC	F: TTTGAAATCGGCTCAGGAAAA	MLSB
	R: ATGGTCTATTTCAATGGCAGTTACG	
ermF	F: CAGCTTTGGTTGAACATTTACGAA	MLSB
	R: AAATTCCTAAAATCACAACCGACAA	
ermJ/ermD	F: GGACTCGGCAATGGTCAGAA	MLSB
	R: CCCCGAAACGCAATATAATGTT	
ermK-01	F: GTTTGATATTGGCATTGTCAGAGAAA	MLSB
	R: ACCATTGCCGAGTCCACTTT	
ermK-02	F: GAGCCGCAAGCCCCTTT	MLSB
	R: GTGTTTCATTTGACGCGGAGTAA	
ermT-01	F: GTTCACTAGCACTATTTTTAATGACAGAAGT	MLSB
	R: GAAGGGTGTCTTTTTAATACAATTAACGA	
ermT-02	F: GTAAAATCCCTAGAGAATACTTTCATCCA	MLSB
	R: TGAGTGATATTTTTGAAGGGTGTCTT	
ermX	F: GCTCAGTGGTCCCCATGGT	MLSB
	R: ATCCCCCCGTCAACGTTT	
ermY	F: TTGTCTTTGAAAGTGAAGCAACAGT	MLSB
	R: TAACGCTAGAGAACGATTTGTATTGAG	
floR	F: ATTGTCTTCACGGTGTCCGTTA	FCA
	R: CCGCGATGTCGTCGAACT	
folA	F: CGAGCAGTTCCTGCCAAAG	other/efflux
	R: CCCAGTCATCCGGTTCATAATC	
fox5	F: GGTTTGCCGCTGCAGTTC	Beta Lactamase
	R: GCGGCCAGGTGACCAA	
intI1	F: CGAACGAGTGGCGGAGGGTG	MGE
	R: TACCCGAGAGCTTGGCACCCA	
IS613	F: AGGTTCGGACTCAATGCAACA	MLSB
	R: TTCAGCACATACCGCCTTGAT	
lmrA-01	F: TCGACGTGACCGTAGTGAACA	MLSB
	R: CGTGACTACCCAGGTGAGTTGA	
lmrA-02	F: TTCAGATGCAATGGCGTTTG	MLSB

	R: ATAATCGGGAACATAATGAGCATAACTAC	
lnuA-01	F: TGACGCTCAACACACTCAAAAA	MLSB
	R: TTCATGCTTAAGTTCCATACGTGAA	
lnuA-02	F: AGAATGAAAAAGAAGCTGAGCTTCTT	MLSB
	R: AAGGTGGCAATTACGTTTTTCAAA	
lnuB-01	F: TGAACATAATCCCCTCGTTTAAAGAT	MLSB
	R: TAATTGCCCTGTTTCATCGTAAATAA	
lnuB-02	F: AAAGGAGAAGGTGACCAATACTCTGA	MLSB
	R: GGAGCTACGTCAAACAACCAGTT	
lnuC	F: TGGTCAATATAACAGATGTAAACCAGATTT	MLSB
	R: CACCCCAGCCACCATCAA	
matA/mel	F: TAGTAGGCAAGCTCGGTGTTGA	MLSB
	R: CCTGTGCTATTTTAAGCCTTGTTTCT	
mdtA	F: CCTAACGGGCGTGACTTCA	MLSB
	R: TTCACCTGTTTCAAGGGTCAAA	
mecA	F: GGTTACGGACAAGGTGAAATACTGAT	Beta Lactamase
	R: TGTCTTTTAATAAGTGAGGTGCGTTAATA	
mefA	F: CCGTAGCATTGGAACAGCTTTT	MLSB
	R: AAACGGAGTATAAGAGTGCTGCAA	
mexA	F: AGGACAACGCTATGCAACGAA	FCA
	R: CCGGAAAGGGCCGAAAT	
mexD	F: TTGCCACTGGCTTTCATGAG	FCA
	R: CACTGCGGAGAACTGTCTGTAGA	
mexE	F: GGTCAGCACCGACAAGGTCTAC	FCA
	R: AGCTCGACGTACTTGAGGAACAC	
mexF	F: CCGCGAGAAGGCCAAGA	FCA
	R: TTGAGTTCGGCGGTGATGA	
mphA-01	F: CTGACGCGCTCCGTGTT	MLSB
	R: GGTGGTGCATGGCGATCT	
mphA-02	F: TGATGACCCTGCCATCGA	MLSB
	R: TTCGCGAGCCCCTCTTC	
mphB	F: CGCAGCGCTTGATCTTGTAG	MLSB
	R: TTACTGCATCCATACGCTGCTT	
mphC	F: CGTTTGAAGTACCGAATTGGAAA	MLSB
	R: GCTGCGGGTTTGCCTGTA	
msrA-01	F: CTGCTAACACAAGTACGATTCCAAAT	MLSB
	R: TCAAGTAAAGTTGTCTTACCTACACCATT	
msrA-02	F: AACGAAATCAAGCGCAACAA	MLSB
	R: CAACCGTGCCTTTTTCTTTTG	
msrC-01	F: TCAGACCGGATCGGTTGTC	MLSB
	R: CCTATTTTTTGGAGTCTTCTCTCTAATGTT	
msrC-02	F: GAATCACTTGTCCGCAGTTTGTT	MLSB
	R: CGTACACAACGGTTTCGTCAGA	
NDM1	F: ATTAGCCGCTGCATTGAT	Beta Lactamase

	R: CATGTCGAGATAGGAAGTG	
oleC	F: CCCGGAGTCGATGTTCGA<br>R: GCCGAAGACGTACACGAACAG	MLSB
oprJ	F: ACGAGAGTGGCGTCGACAA<br>R: AAGGCGATCTCGTTGAGGAA	FCA
pbp	F: CCGGTGCCATTGGTTTAGA<br>R: AAAATAGCCGCCCCAAGATT	Beta Lactamase
pbp2x	F: TTTCATAAGTATCTGGACATGGAAGAA<br>R: CCAAAGGAAACTTGCTTGAGATTAG	Beta Lactamase
Pbp5	F: GGCGAACTTCTAATTAATCCTATCCA<br>R: CGCCGATGACATTCTTCTTATCTT	Beta Lactamase
penA	F: AGACGGTAACGTATAACTTTTTGAAAGA<br>R: GCGTGTAGCCGGCAATG	Beta Lactamase
pikR1	F: TCGACATGCGTGACGAGATT<br>R: CCGCGAATTAGGCCAGAA	MLSB
pikR2	F: TCGTGGGCCAGGTGAAGA<br>R: TTCCCCTTGCCGGTGAA	MLSB
pmrA	F: TTTGCAGGTTTTGTTCCTAATGC<br>R: GCAGAGCCTGATTTCTCCTTTG	FCA
qnrA	F: AGAGGATTTCTCACGCCAGG<br>R: TGCCAGGCACAGATCTTGAC	FCA
spcN-01	F: AAAAGTTCGATGAAACACGCCTAT<br>R: TCCAGTGGTAGTCCCCGAATC	Aminoglycosid e
spcN-02	F: CAGAATCTTCCTGAAAAGTTTGATGAA<br>R: CGCAGACACGCCGAATC	Aminoglycosid e
str	F: AATGAGTTTTGGAGTGTCTCAACGTA<br>R: AATCAAAACCCCTATTAAAGCCAAT	Aminoglycosid e
strA	F: CCGGTGGCATTTGAGAAAAA<br>R: GTGGCTCAACCTGCGAAAAG	Aminoglycosid e
strB	F: GCTCGGTCGTGAGAACAATCT<br>R: CAATTTCGGTCGCCTGGTAGT	Aminoglycosid e
sul1	F: CACCGGAAACATCGCTGCA<br>R: AAGTTCCGCCGCAAGGCT	Sulfonamide
sul2	F: GTCAAAGAACGCCGCAATGT<br>R: TCATCTGCCAAACTCGTCGTTA	Sulfonamide
sulA/folP-01	F: CAGGCTCGTAAATTGATAGCAGAAG<br>R: CTTTCCTTGCGAATCGCTTT	Sulfonamide
sulA/folP-02	F: GCGATTCGCAAGGAAAGTGA<br>R: CACATGGGCCATTTTTTCATC	Sulfonamide

sulA/folP-03	F: CACGGCTTCGGCTCATGT	Sulfonamide
	R: TGCCATCCTGTGACTAGCTACGT	
tet(32)	F: CCATTACTTCGGACAACGGTAGA	Tetracycline
	R: CAATCTCTGTGAGGGCATTTAACA	
tet(34)	F: CTTAGCGCAAACAGCAATCAGT	Tetracycline
	R: CGGTGATACAGCGCGTAAACT	
tet(35)	F: ACCCCATGACGTACCTGTAGAGA	Tetracycline
	R: CAACCCACACTGGCTACCAGTT	
tet(36)-01	F: AGAATACTCAGCAGAGGTCAGTTCCT	Tetracycline
	R: TGGTAGGTCGATAACCCGAAAAT	
tet(36)-02	F: TGCAGGAAAGACCTCCATTACAG	Tetracycline
	R: CTTTGTCCACACTTCCACGTACTATG	
tet(37)	F: GAGAACGTTGAAAAGGTGGTGAA	Tetracycline
	R: AACCAAGCCTGGATCAGTCTCA	
tet(38)	F: TTAATGTGGCGGTATCTGTAGGTATT	Tetracycline
	R: TTGCCTGGGAAATTTAATGCTTT	
tetA-01	F: GCTGTTTGTTCTGCCGGAAA	Tetracycline
	R: GGTTAAGTTCCTTGAACGCAAACT	
tetA-02	F: CTCACCAGCCTGACCTCGAT	Tetracycline
	R: CACGTTGTTATAGAAGCCGCATAG	
tetB-01	F: AGTGCGCTTTGGATGCTGTA	Tetracycline
	R: AGCCCCAGTAGCTCCTGTGA	
tetB-02	F: GCCCAGTGCTGTTGTTGTCAT	Tetracycline
	R: TGAAAGCAAACGGCCTAAATACA	
tetC-01	F: CATATCGCAATACATGCGAAAAA	Tetracycline
	R: AAAGCCGCGGTAAATAGCAA	
tetC-02	F: ACTGGTAAGGTAAACGCCATTGTC	Tetracycline
	R: ATGCATAAACCAGCCATTGAGTAAG	
tetD-01	F: TGCCGCGTTTGATTACACA	Tetracycline
	R: CACCAGTGATCCCGGAGATAA	
tetD-02	F: TGTCATCGCGCTGGTGATT	Tetracycline
	R: CATCCGCTTCCGGGAGAT	
tetE	F: TTGGCGCTGTATGCAATGAT	Tetracycline
	R: CGACGACCTATGCGATCTGA	
tetG-01	F: TCAACCATTGCCGATTCGA	Tetracycline
	R: TGGCCCGGCAATCATG	
tetG-02	F: CATCAGCGCCGGTCTTATG	Tetracycline
	R: CCCCATGTAGCCGAACCA	
tetH	F: TTTGGGTCATCTTACCAGCATTAA	Tetracycline
	R: TTGCGCATTATCATCGACAGA	
tetJ	F: GGGTGCCGCATTAGATTACCT	Tetracycline
	R: TCGTCCAATGTAGAGCATCCATA	
tetK	F: CAGCAGTCATTGGAAAATTATCTGATTATA	Tetracycline
	R: CCTTGTACTAACCTACCAAAAATCAAAATA	

tetL-01	F: AGCCCGATTTATTCAAGGAATTG<br>R: CAAATGCTTTCCCCCTGTTCT	Tetracycline
tetL-02	F: ATGGTTGTAGTTGCGCGCTATAT<br>R: ATCGCTGGACCGACTCCTT	Tetracycline
tetM-01	F: CATCATAGACACGCCAGGACATAT<br>R: CGCCATCTTTTGCAGAAATCA	Tetracycline
tetM-02	F: TAATATTGGAGTTTTAGCTCATGTTGATG<br>R: CCTCTCTGACGTTCTAAAAGCGTATTAT	Tetracycline
tetO-01	F: ATGTGGATACTACAACGCATGAGATT<br>R: TGCCTCCACATGATATTTTTCCT	Tetracycline
tetO-02	F: CAACATTAACGGAAAGTTTATTGTATACCA<br>R: TTGACGCTCCAAATTCATTGTATC	Tetracycline
tetPA	F:<br>AGTTGCAGATGTGTATAGTCGTAAACTATCTAT<br>T<br>R: TGCTACAAGTACGAAAACAAAACTAGAA	Tetracycline
tetPB-01	F: ACACCTGGACACGCTGATTTT<br>R: ACCGTCTAGAACGCGGAATG	Tetracycline
tetPB-02	F: TGATACACCTGGACACGCTGAT<br>R: GTCCAAAACGCGGAATG	Tetracycline
tetPB-03	F: TGGGCGACAGTAGGCTTAGAA<br>R: TGACCCTACTGAAACATTAGAAATATACCT	Tetracycline
tetPB-04	F: AGTGGTGCAAATACTGAAAAAGTTGT<br>R: TTTGTTCCTTCGTTTTGGACAGA	Tetracycline
tetPB-05	F: CTGAAGTGGAGCGATCATTCC<br>R: CCCTCAACGGCAGAAATAACTAA	Tetracycline
tetQ	F: CGCCTCAGAAGTAAGTTCATACACTAAG<br>R: TCGTTCATGCGGATATTATCAGAAT	Tetracycline
tetR-01	F: ATGAGTTCGGCCAGAATTTCC<br>R: GGTTGTGCGCGAAATGATT	Tetracycline
tetR-02	F: CGCGATAGACGCCTTCGA<br>R: TCCTGACAACGAGCCTCCTT	Tetracycline
tetR-03	F: CGCGATGGAGCAAAAGTACAT<br>R: AGTGAAAAACCTTGTTGGCATAAAA	Tetracycline
tetS	F: TTAAGGACAAACTTTCTGACGACATC<br>R: TGTCTCCCATTGTTCTGGTTCA	Tetracycline
tetT	F: CCATATAGAGGTTCCACCAAATCC<br>R: TGACCCTATTGGTAGTGGTTCTATTG	Tetracycline
tetU-01	F: GTGGCAAAGCAACGGATTG<br>R: TGCGGGCTTGCAAAACTATC	Tetracycline
tetU-02	F: AACAGCGGGTTAAGTGTGCAA<br>R: ATGGTATCATTCAGTTTTCCGACAAT	Tetracycline
tetV	F: GCGGGAACGACGATGTATATC<br>R: CCGCTATCTCACGACCATGAT	Tetracycline

tetW-01	F: ATGAACATTCCCACCGTTATCTTT<br>R: ATATCGGCGGAGAGCTTATCC	Tetracycline
tetX	F: AAATTTGTTACCGACACGGAAGTT<br>R: CATAGCTGAAAAAATCCAGGACAGTT	Tetracycline
tnpA-01	F: AATTGATGCGGACGGCTTAA<br>R: TCACCAAACTGTTTATGGAGTCGTT	MGE
tnpA-02	F: CATCATCGGACGGACAGAATT<br>R: GTCGGAGATGTGGGTGTAGAAAGT	MGE
tnpA-03	F: GGGCGGGTCGATTGAAA<br>R: GTGGGCGGGATCTGCTT	MGE
tnpA-04	F: CCGATCACGGAAAGCTCAAG<br>R: GGCTCGCATGACTTCGAATC	MGE
tnpA-05	F: GAAACCGATGCTACAATATCCAATTT<br>R: CAGCACCGTTTGCAGTGTAAG	MGE
tnpA-07	F: TGCAGATGGTTTAACCTTGGATATTT<br>R: TCGGTTCATCAAACTGCTTCAC	MGE
vanA	F: AAAAGGCTCTGAAAACGCAGTTAT<br>R: CGGCCGTTATCTTGTAAAAACAT	Vancomycin
vanB-01	F: TTGTCGGCGAAGTGGATCA<br>R: AGCCTTTTTCCGGCTCGTT	Vancomycin
vanB-02	F: CCGGTCGAGGAACGAAATC<br>R: TCCTCCTGCAAAAAAAGATCAAC	Vancomycin
vanC-01	F: ACAGGGATTGGCTATGAACCAT<br>R: TGACTGGCGATGATTTGACTATG	Vancomycin
vanC-02	F: CCTGCCACAATCGATCGTT<br>R: CGGCTTCATTCGGCTTGATA	Vancomycin
vanC-03	F: AAATCAATACTATGCCGGGCTTT<br>R: CCGACCGCTGCCATCA	Vancomycin
vanC1	F: AGGCGATAGCGGGTATTGAA<br>R: CAATCGTCAATTGCTCATTTCC	Vancomycin
vanC2/vanC3	F: TTTGACTGTCGGTGCTTGTGA<br>R: TCAATCGTTTCAGGCAATGG	Vancomycin
vanD	F: CAGAGGAACATAATGTTTCGATAAAATCT<br>R: GCCGGATTTTGTGATTCCAA	Vancomycin
vanG	F: ATTTGAATTGGCAGGTATACAGGTTA<br>R: TGATTTGTCTTTGTCCATACATAATGC	Vancomycin
vanHB	F: GAGGTTTCCGAGGCGACAA<br>R: CTCTCGGCGGCAGTCGTAT	Vancomycin
vanHD	F: GTGGCCGATTATACCGTCATG<br>R: CGCAGGTCATTCAGGCAAT	Vancomycin
vanRA-01	F: CCCTTACTCCCACCGAGTTTT<br>R: TTCGTCGCCCCATATCTCAT	Vancomycin
vanRA-02	F: CCACTCCGGCCTTGTCATT<br>R: GCTAACCACATTCCCCTTGTTTT	Vancomycin

vanRB	F: GCCCTGTCGGATGACGAA<br>R: TTACATAGTCGTCTGCCTCTGCAT	Vancomycin
vanRC	F: TGCGGGAAAAACTGAACGA<br>R: CCCCCCATACGGTTTTGATTA	Vancomycin
vanRC4	F: AGTGCTTTGGCTTATCTCGAAAA<br>R: TCCGGCAGCATCACATCTAA	Vancomycin
vanRD	F: TTATAATGGCAAGGATGCACTAAAGT<br>R: CGTCTACATCCGGAAGCATGA	Vancomycin
vanSA	F: CGCGTCATGCTTTCAAAATTC<br>R: TCCGCAGAAAGCTCAATTTGTT	Vancomycin
vanSB	F: GCGCGGCAAATGACAAC<br>R: TTTGCCATTTTATTCGCACTGT	Vancomycin
vanSC-01	F: ATCAACTGCGGGAGAAAAGTCT<br>R: TCCGCTGTTCCGCTTCTT	Vancomycin
vanSC-02	F: GCCATCAGCGAGTCTGATGA<br>R: CAGCTGGGATCGTTTTTCCTT	Vancomycin
vanSE	F: TGGCCGAAGAAGCAGGAA<br>R: CAATAATACTCGTCAAAGGAGTTCTCA	Vancomycin
vanTC-01	F: CACACGCATTTTTTCCCATCTAG<br>R: CAGCCAACAGATCATCAAAACAA	Vancomycin
vanTC-02	F: ACAGTTGCCGCTGGTGAAG<br>R: CGTGGCTGGTCGATCAAAA	Vancomycin
vanTE	F: GTGGTGCCAAGGAAGTTGCT<br>R: CGTAGCCACCGCAAAAAAAT	Vancomycin
vanTG	F: CGTGTAGCCGTTCCGTTCTT<br>R: CGGCATTACAGGTATATCTGGAAA	Vancomycin
vanWB	F: CGGACAAAGATACCCCCTATAAAG<br>R: AAATAGTAAATTGCTCATCTGGCACAT	Vancomycin
vanWG	F: ACATTTTCATTTTGGCAGCTTGTAC<br>R: CCGCCATAAGAGCCTACAATCT	Vancomycin
vanXA	F: CGCTAAATATGCCACTTGGGATA<br>R: TCAAAAGCGATTCAGCCAACT	Vancomycin
vanXB	F: AGGCACAAAATCGAAGATGCTT<br>R: GGGTATGGCTCATCAATCAACTT	Vancomycin
vanXD	F: TAAACCGTGTTATGGGAACGAA<br>R: GCGATAGCCGTCCCATAAGA	Vancomycin
vanYB	F: GGCTAAAGCGGAAGCAGAAA<br>R: GATATCCACAGCAAGACCAAGCT	Vancomycin
vanYD-01	F: AAGGCGATACCCTGACTGTCA<br>R: ATTGCCGGACGGAAGCA	Vancomycin
vanYD-02	F: CAAACGGAAGAGAGGTCACTTACA<br>R: CGGACGGTAATAGGGACTGTTC	MLSB
vatB-01	F: GGAAAAAGCAACTCCATCTCTTGA<br>R: TCCTGGCATAACAGTAACATTCTGA	MLSB

vatB-02	F: TTGGGAAAAAGCAACTCCATCT<br>R: CAATCCACACATCATTTCCAACA	MLSB
vatC-01	F: CGGAAATTGGGAACGATGTT<br>R: GCAATAATAGCCCCGTTTCCTA	MLSB
vatC-02	F: CGATGTTTGGATTGGACGAGAT<br>R: GCTGCAATAATAGCCCCGTTT	MLSB
vatD	F: TGCAATAGTAGCTGCTAATTCTGTTGTT<br>R: TGTTTTATTTCGTTAGCAGGATTTCC	MLSB
vatE-01	F: GGTGCCATTATCGGAGCAAAT<br>R: TTGGATTGCCACCGACAAT	MLSB
vatE-02	F: GACCGTCCTACCAGGCGTAA<br>R: TTGGATTGCCACCGACAATT	MLSB
vgaA-01	F: CGAGTATTGTGGAAAGCAGCTAGTT<br>R: CCCGTACCGTTAGAGCCGATA	MLSB
vgaA-02	F: GACGGGTATTGTGGAAAGCAA<br>R: TTTCCTGTACCATTAGATCCGATAATT	MLSB
vgaB-01	F: TAAAAGAGAATAAGGCGCAAGGA<br>R: TGTTTAGTAGCATGTTGCATTTTCC	MLSB
vgaB-02	F: GAATGATTAAGCCCCCTTCAAAA<br>R: ATTCGTGTTTCCAACGATTTCG	MLSB
vgb-01	F: AGGGAGGGTATCCATGCAGAT<br>R: ACCAAATGCGCCCGTTT	MLSB
vgb-02	F: CCACGATGGCTGCCTTTG<br>R: GGCCATGCAGGACGGATAT	MLSB
vgbB-01	F: ATACGAGCTGCCTAATAAAGGATCTT<br>R: TGTGAACCACAGGGCATTATCA	MLSB
vgbB-02	F: CAGCCGGATTCTGGTCCTT<br>R: TACGATCTCCATTCAATTGGGTAAA	MLSB
yidY/mdtL-01	F: GCAGTTGCATATCGCCTTCTC<br>R: CTTCCCGGCAAACAGCAT	FCA
yidY/mdtL-02	F: TGCTGATCGGGATTCTGATTG<br>R: CAGGCGCGACGAACATAAT	FCA

Table S4. The relative abundance of antibiotic resistance genes and mobile genetic elements.

Classification	Genes	C1	E1	E2	E3	E4	Total
Aminoglycoside	*SPCN-01*				5.27E-07		0.000000527
Aminoglycoside	*SPCN-02*		1.09E-05				0.0000109
Aminoglycoside	*STR*	0.0000858	0.00000602	0.000143595	3.50E-05	0.000163918	0.000434333
Aminoglycoside	*STRA*	0.001743155	0.003668407	0.009668469	0.01639573	0.000240347	0.031716108

Aminoglycoside	*STRB*	0.004258684	0.023000709	0.015810569	0.017815625	0.001286669	0.062172256
Aminoglycoside	*APHA3-01*	0.15288264	0.173284235	0.033885432	0.261696252	0.251208721	0.87295728
Aminoglycoside	*APHA3-02*	0.149145486	0.187338574	0.041122992	0.279687293	0.266459193	0.923753538
Aminoglycoside	*APHA1*	0.023240114	0.026311127	0.017531195	0.027914039	0.000397702	0.095394177
Aminoglycoside	*APH(2')-ID-01*	9.27E-05	0.000366079	0.000158442	0.002753184	0.00017494	0.003545345
Aminoglycoside	*APH(2')-ID-02*	3.34E-05	0.000236879	6.84E-05	0.000966913	4.24E-06	0.001309832
Aminoglycoside	*AADD*	0.058791452	0.009668673	0.017380231	0.044063392	0.05958792	0.189491668
Aminoglycoside	*AADE*	0.056740114	0.018887247	0.041152085	0.037112732	0.004497068	0.158389246
Aminoglycoside	*AAC*				8.26E-06	1.82E-07	0.000008442
Aminoglycoside	*AAC(6')-IB-1*	3.99E-05	0.001316289	0.000359472	0.001510936	5.10E-05	0.003277597
Aminoglycoside	*AAC(6')-IB-2*	6.06E-05	0.001385479	0.000450854	0.0017405	6.30E-05	0.003700433
Aminoglycoside	*AAC(6')-IB-3*	6.40E-05	0.002271616	0.000637036	0.002136072	8.40E-05	0.005192724
Aminoglycoside	*AAC(6')-II*	3.49E-05	0.00023671	2.35E-05	4.18E-05	1.03E-05	0.00034721
Aminoglycoside	*AAC(6')-IY*	3.26E-06	4.47E-07		2.12E-05		0.000024907
Aminoglycoside	*AACA/APHD*	0.083245637	0.045307549	0.351425974	0.276497149	0.662687464	1.419163773
Aminoglycoside	*AACC*				8.26E-06	1.82E-07	0.000008442
Aminoglycoside	*AACC2*	0.002504346	0.001931972	0.001158877	0.000643498	9.17E-06	0.006247863
Aminoglycoside	*AACC4*	0.003434437	0.002459266	0.003562095	0.007142115	3.26E-05	0.016630513
Aminoglycoside	*AADA-01*	0.027781738	0.016698168	0.02536559	0.061569256	0.000148103	0.131562855
Aminoglycoside	*AADA-02*	0.034532243	0.020562666	0.032244141	0.072129026	0.000169468	0.159637544
Aminoglycoside	*AADA1*	0.023693378	0.023433098	0.02550901	0.069009403	0.00020332	0.141848209
Aminoglycoside	*AADA-1-01*	0.037275395	0.052254241	0.012377345	0.108951344	0.065743483	0.276601808

Aminoglycoside	*AADA-1-02*	0.131332576	0.146099881	0.028262536	0.236278555	0.219147116	0.761120664
Aminoglycoside	*AADA2-01*	0.000861201	0.010890198	0.00356738	0.016826966	5.41E-05	0.032199845
Aminoglycoside	*AADA2-02*	0.000237957	0.001997034	0.000794714	0.003255243	1.15E-05	0.006296448
Aminoglycoside	*AADA2-03*	0.025975819	0.030843	0.027463058	0.075309163	0.000263937	0.159854977
Aminoglycoside	*AADA5-01*	6.65E-05	0.001484199	0.000155812	0.000558782	2.20E-05	0.002287293
Aminoglycoside	*AADA5-02*	3.25E-05	0.000515037	8.91E-05	0.000329397	1.25E-05	0.000978534
Aminoglycoside	*AADA9-01*	1.45E-06				8.80E-07	0.00000233
Aminoglycoside	*AADA9-02*					9.31E-07	0.000000931
Beta Lactamase	*AMPC/BLADHA*	1.30E-05	0.000147164	0.000357876	0.001770361	3.23E-05	0.002320701
Beta Lactamase	*AMPC-01*	8.70E-06	8.13E-06	3.17E-06	1.43E-05	4.86E-07	0.000034786
Beta Lactamase	*AMPC-02*	0.010856441	0.012924041	0.011086799	0.019578005	0.00035799	0.054803276
Beta Lactamase	*AMPC-03*	0.019804419	0.037214798	0.008788495	0.031671965	0.000596286	0.098075963
Beta Lactamase	*AMPC-04*	3.90E-06					0.0000039
Beta Lactamase	*AMPC-06*	2.61E-06	1.78E-05				0.00002041
Beta Lactamase	*BLA1*					2.57E-06	0.00000257
Beta Lactamase	*BLACMY*	9.27E-06		0.001751136	0.000148383	1.61E-06	0.001910399
Beta Lactamase	*BLACMY2-01*	0.000177109	0.000295929	0.001121885	0.000249716	1.01E-06	0.001845649
Beta Lactamase	*BLACMY2-02*	7.46E-08	7.08E-07	2.31E-06	3.02E-07	9.45E-07	4.3396E-06
Beta Lactamase	*BLACTX-M-01*	4.95E-05	0.000577723	0.000102749	0.001528955	1.32E-05	0.002272127
Beta Lactamase	*BLACTX-M-02*	0.000713002	0.007462712	0.001281029	0.014439877	0.000161106	0.024057726
Beta Lactamase	*BLACTX-M-03*	0.025680578	0.019877221	0.009879958	0.004972223	0.000504419	0.060914399
Beta Lactamase	*BLACTX-M-04*		3.87E-07				0.000000387
Beta Lactamase	*BLACTX-M-05*				5.04E-07		0.000000504
Beta Lactamase	*BLACTX-M-06*	0.006653184	0.00557016	0.002850521	0.001320742	9.43E-05	0.016488907

Beta Lactamase	*BLAGES*			2.72E-06	4.30E-06		0.00000702
Beta Lactamase	*BLAIMP-01*	2.39E-08				1.42E-07	1.659E-07
Beta Lactamase	*BLAIMP-02*				3.01E-06		0.00000301
Beta Lactamase	*BLA-L1*		2.53E-06		2.00E-07	1.15E-07	0.000002845
Beta Lactamase	*BLAMOX/BLACMY*	1.44E-06		1.15E-06	4.63E-06	5.57E-07	0.000007777
Beta Lactamase	*BLAOXA1/BLAOXA30*	0.000108957	0.002424381	0.00049227	0.002083641	2.57E-05	0.005134949
Beta Lactamase	*BLAOXA10-01*	0.007527156	0.003364996	0.009212549	0.017483581	7.10E-06	0.037595382
Beta Lactamase	*BLAOXA10-02*	0.000298726	0.000165296	0.000420187	0.000749865	2.64E-08	0.0016341
Beta Lactamase	*BLAPER*	1.89E-06		3.26E-06	1.72E-05	2.16E-07	0.000022566
Beta Lactamase	*BLAPSE*	2.67E-06	0.000193315	2.09E-05	3.56E-05	2.03E-06	0.000254515
Beta Lactamase	*BLAROB*		4.70513E-08				4.70513E-08
Beta Lactamase	*BLASFO*	3.51E-06	8.99E-06	2.52E-06	2.64E-05	9.95E-07	0.000042415
Beta Lactamase	*BLASHV-01*	0.000180393	1.99E-06	8.31E-06	3.94E-06		0.000194633
Beta Lactamase	*BLASHV-02*	0.000480506	2.75E-06		2.99E-06		0.000486246
Beta Lactamase	*BLATEM*	0.008108266	0.01400033	0.013782974	0.020125554	0.00029751	0.056314634
Beta Lactamase	*BLAVEB*	1.81E-06	9.55E-06	6.90E-06		5.61E-07	0.000018821
Beta Lactamase	*BLAVIM*	2.17E-06			3.61E-05	5.29E-07	0.000038799
Beta Lactamase	*BLAZ*	9.58E-05	8.97E-05	5.55E-05	7.76E-05	8.03E-06	0.00032663
Beta Lactamase	*CEPA*		6.16E-06	5.25E-06	0.000167298	3.24E-05	0.000211108
Beta Lactamase	*CFIA*	3.57E-05	9.98E-05	1.02E-05	0.001704606	1.94E-05	0.001869706
Beta Lactamase	*CFXA*	0.000776743	0.02924573	0.00141941	0.01385969	0.001153551	0.046455124
Beta Lactamase	*FOX5*			4.22E-05	6.42E-05	1.33E-06	0.00010773
Beta Lactamase	*NDM1*	4.07E-06	0.006192254	0.002339188	0.022359601	1.59E-06	0.030896703
Beta Lactamase	*PBP*		4.89E-07				0.000000489

Beta Lactamase	*PBP5*	0.003096863	0.008894399	0.000436444	0.000724564	0.000383402	0.013535672
FCA	*ACRA-01*	0.006824532	0.010311195	0.007596853	0.013187125	0.000236996	0.038156701
FCA	*ACRA-02*	0.008498565	0.010430208	0.008935615	0.015903157	6.76E-05	0.043835145
FCA	*ACRA-05*	2.62E-06	1.14E-05	3.65E-06	1.72E-05	9.60E-07	0.00003583
FCA	*ACRB-01*	0.007572437	0.011749189	0.008152194	0.014649063	0.000267488	0.042390371
FCA	*ACRB-02*	9.89E-07			2.14E-05		0.000022389
FCA	*ACRF*	0.008897789	0.015517278	0.008347198	0.013062703	0.000425903	0.046250871
FCA	*ADEA*					7.77E-08	7.77E-08
FCA	*CATA1*	1.79E-06	7.14E-06	0.001121828	7.76E-05	6.23E-07	0.001208981
FCA	*CATB3*	8.73E-06	0.001270975	0.000260998	0.001193319	1.46E-05	0.002748622
FCA	*CFR*		9.60E-07				0.00000096
FCA	*CMEA*	0.004170639	0.000338751	0.001440711	5.66E-07	2.04E-05	0.005971067
FCA	*CMLA1-01*	0.00341656	0.006120455	0.005256255	0.014834239	1.53E-05	0.029642809
FCA	*CMLA1-02*	0.005056634	0.008783057	0.007358992	0.020530348	3.03E-05	0.041759331
FCA	*CMX(A)*	0.000807126	6.97E-05	0.000228607	0.001206664	4.94E-05	0.002361497
FCA	*EMRB/QACA*				7.03E-06		0.00000703
FCA	*FLOR*	0.016842863	0.019225839	0.012493243	0.021593737	0.000336073	0.070491755
FCA	*MEXA*		2.79E-06	2.51E-06	2.41E-06	3.72E-07	0.000008082
FCA	*MEXD*		3.32E-06	5.94E-08			3.3794E-06
FCA	*MEXE*	3.27E-06	1.84E-06	2.02E-06		1.01E-07	0.000007231
FCA	*MEXF*	0.000248423	0.001093969	0.000523503	0.002079422	0.000181635	0.004126952
FCA	*QNRA*		1.10E-05		7.75E-07	4.85E-07	0.00001226
FCA	*YIDY/MDTL-01*	2.25E-07	2.30E-07	5.29E-07	2.78E-05	5.94E-08	2.88434E-05
FCA	*YIDY/MDTL-02*	0.01434078	0.02170682	0.01459472	0.022370623	0.000404758	0.073417701
MLSB	*CARB*				3.71E-06		0.00000371

MLSB	*EREA*	4.20E-07	6.53E-05	1.65E-05	2.43E-05	6.68E-07	0.000107188
MLSB	*ERM(35)*	0.000292704	0.000192291	0.000149115	0.00013456	2.72E-05	0.00079587
MLSB	*ERM(36)*	4.83E-05	2.42E-05	2.63E-05	3.81E-05	3.22E-06	0.00014012
MLSB	*ERMA*	9.69E-05	5.53E-05	3.82E-05	9.85E-05	8.29E-06	0.00029719
MLSB	*ERMB*	0.794180604[a]	0.47366172	0.23266149[b]	0.37524189[b]	0.436219689	2.311965411
MLSB	*ERMC*	0.013513596	0.005862423	0.009673163	0.020766814	0.002273366	0.052089362
MLSB	*ERMF*	0.000999906	0.031985052	0.002379579	0.056206922	0.003746037	0.095317496
MLSB	*ERMJ/ERMD*				4.20E-06		0.0000042
MLSB	*ERMK-01*				5.42E-06		0.00000542
MLSB	*ERMT-01*	0.311396133	0.296998129	0.135426424	0.129181414	0.474189678	1.347191778
MLSB	*ERMT-02*	0.282804539	0.27273199	0.128670817	0.119965168	0.423741374	1.227913888
MLSB	*ERMX*	2.70E-05	2.92E-05	7.05E-05	0.000528739	2.19E-05	0.000677339
MLSB	*LNUA-01*	0.03768124	0.040886965	0.006618785	0.030143769	0.049060238	0.164390997
MLSB	*LNUB-01*	0.001819704	0.000691495	0.000681966	0.000379426	0.000547734	0.004120325
MLSB	*LNUB-02*	0.001338414	0.000485005	0.000362287	0.000209754	0.000476104	0.002871564
MLSB	*LNUC*	7.22E-07	7.06E-07	1.42E-07	1.44E-06	5.96E-08	3.0696E-06
MLSB	*MATA/MEL*	0.000729031	0.000631444	0.000308496	0.000530218	3.96E-05	0.002238789
MLSB	*MEFA*	0.001558619	0.013640432	0.000465191	0.012797242	0.000199685	0.028661169
MLSB	*MPHA-01*	0.006678079	0.004444522	0.002232994	0.006869916	5.20E-05	0.020277511
MLSB	*MPHA-02*	0.003658464	0.00312218	0.001499625	0.004264197	3.29E-05	0.012577366
MLSB	*MPHB*		2.00E-06				0.000002
MLSB	*MSRA-01*			4.75E-06	6.21E-06	1.96E-07	0.000011156
MLSB	*MSRA-02*	3.40E-06	2.92E-06	3.40E-06	1.74E-06	5.93E-07	0.000012053
MLSB	*MSRC-01*	2.95E-08			1.59E-05	8.24E-07	1.67535E-05
MLSB	*VATB-01*				2.83E-06	9.86E-07	0.000003816

MLSB	*VATB-02*	1.26E-05	1.97E-06		2.17E-06	1.66E-07	0.000016906
MLSB	*VATD*		3.29E-08	1.17E-07		3.57E-08	1.856E-07
MLSB	*VATE-01*	0.006355161	0.00266915	0.011900622	0.044725857	0.004196201	0.069846991
MLSB	*VATE-02*	0.004049666	0.001573539	0.005789017	0.018717536	0.002393553	0.032523311
MLSB	*VGAA-01*		1.99E-06				0.00000199
MLSB	*VGAA-02*	1.67E-05					0.0000167
MLSB	*VGB-01*	8.07E-06	7.64E-06	2.49E-06	3.26E-06	9.72E-07	0.000022432
MLSB	*VGB-02*			9.82E-08	5.64E-07	6.73E-08	7.295E-07
Sulfonamide	*SUL1*	0.001431765	0.003643579	0.001247129	0.009378768	8.96E-05	0.015790841
Sulfonamide	*SUL2*	0.007592505	0.02817112	0.00937318	0.008645936	0.001478583	0.055261324
Tetracycline	*TET(32)*	0.000369704	0.00560579	0.000885576	0.007214519	0.000659267	0.014734856
Tetracycline	*TET(36)-01*			7.36E-07	1.77E-06	2.55E-06	0.000005056
Tetracycline	*TET(36)-02*				3.23E-07	2.37E-07	0.00000056
Tetracycline	*TET(37)*			6.78E-07	3.49E-06	1.53E-08	4.1833E-06
Tetracycline	*TETA-01*	0.01984462	0.028154224	0.021116155	0.04101144	0.000575833	0.110702272
Tetracycline	*TETA-02*	0.017069654	0.026928966	0.018101914	0.033970914	0.000450858	0.096522306
Tetracycline	*TETB-01*	8.21E-06	7.38E-05	0.000571386	0.000281596	2.08E-05	0.000955792
Tetracycline	*TETB-02*		0.00042748	0.00126004	0.000395863	5.18E-05	0.002135183
Tetracycline	*TETC-01*	3.81E-06	0.000217682	0.000675439	0.000295979	2.00E-05	0.00121291
Tetracycline	*TETC-02*	3.13E-06	7.95E-05	0.000476594	0.000179579	1.37E-05	0.000752503
Tetracycline	*TETD-01*	9.94E-06	3.73E-05	1.74E-06		6.73E-07	0.000049653
Tetracycline	*TETD-02*		4.26E-05				0.0000426
Tetracycline	*TETE*	8.17E-07		1.21E-06	5.44E-06		0.000007467
Tetracycline	*TETG-01*	9.26E-06		0.000121718	5.66E-05	1.02E-05	0.000197778
Tetracycline	*TETG-02*	2.19E-05	1.30E-05	0.000372476	8.34E-05	2.01E-05	0.000510876
Tetracycline	*TETH*	2.09E-06		9.12E-06			0.00001121

Tetracycline	*TETJ*	9.65E-06	3.79E-05	4.82E-06	4.51E-05	2.82E-06	0.00010029
Tetracycline	*TETK*	1.80E-06	5.25E-07			6.82E-07	0.000003007
Tetracycline	*TETL-01*		6.26E-06	4.57E-06			0.00001083
Tetracycline	*TETL-02*	0.097289665	0.090153976	0.035676823	0.069350846	0.148565837	0.441037147
Tetracycline	*TETM-01*	0.137737097	0.096397438	0.041825039	0.091581358	0.18525128	0.552792212
Tetracycline	*TETM-02*	0.117019415	0.079868145	0.025406109	0.064709591	0.155834828	0.442838088
Tetracycline	*TETO-01*	0.010378171	0.013613388	0.008115392	0.009165723	0.00087865	0.042151324
Tetracycline	*TETO-02*	0.269527078	0.141052099	0.086886898	0.010398536	0.001151471	0.509016082
Tetracycline	*TETPA*	0.000315161	2.48E-06	0.009311119	0.000235441	1.02E-06	0.009865221
Tetracycline	*TETPB-01*	4.42E-07	3.64E-06	2.81E-06		1.45E-06	0.000008342
Tetracycline	*TETPB-02*		1.50E-06	6.93E-06		1.91E-06	0.00001034
Tetracycline	*TETPB-03*	0.000426681	5.24E-06	0.012775254	0.000348575	1.44E-06	0.01355719
Tetracycline	*TETPB-04*				1.25E-06		0.00000125
Tetracycline	*TETPB-05*		3.03E-06			1.19E-06	0.00000422
Tetracycline	*TETQ*	0.001374255	0.034052839	0.002962955	0.022395978	0.003103126	0.063889153
Tetracycline	*TETR-01*	0.01183095	0.018784623	0.009144945	0.011294519	0.000281154	0.051336191
Tetracycline	*TETR-02*	0.010582038	0.018448615	0.007818683	0.013378163	0.000249056	0.050476555
Tetracycline	*TETR-03*	8.06E-06	9.53E-05	0.00062869	0.000303996	2.10E-05	0.001057046
Tetracycline	*TETS*	1.02E-05	9.70E-06	2.21E-05	1.69E-05	3.14E-06	0.00006204
Tetracycline	*TETT*	1.52E-06				6.97E-07	0.000002217
Tetracycline	*TETU-01*	2.76E-07			3.44E-06	2.79E-07	0.000003995
Tetracycline	*TETU-02*		2.15E-07		4.36E-06		0.000004575
Tetracycline	*TETV*	7.70E-06	4.30E-06	5.24E-06	6.42E-06	5.96E-07	0.000024256
Tetracycline	*TETW-01*	0.512534441	0.679857059	0.349596856	0.208262474	0.14855458	1.89880541
Tetracycline	*TETX*	0.000243253	0.007690261	0.000405479	8.34E-05	0.000139871	0.008562264

Vancomycin	*VANB-01*	1.46E-06			6.24E-06	3.65E-07	0.000008065
Vancomycin	*VANB-02*	4.64E-06	9.32E-06		8.43E-06	1.17E-07	0.000022507
Vancomycin	*VANC-01*			5.20E-07	2.37E-05		0.00002422
Vancomycin	*VANC-02*	1.42E-06			3.48E-05	7.54E-07	0.000036974
Vancomycin	*VANC-03*	1.58E-06	1.18E-05	2.84E-06	2.91E-05	4.26E-07	0.000045746
Vancomycin	*VANC1*				8.32E-06	3.99E-07	0.000008719
Vancomycin	*VANC2/VANC3*	1.64E-06		4.34E-06	1.31E-05		0.00001908
Vancomycin	*VANRA-01*	2.58E-06		1.80E-06	2.25E-06	5.30E-08	0.000006683
Vancomycin	*VANRB*	2.75E-06	1.37E-05		7.73E-06	1.35E-07	0.000024315
Vancomycin	*VANRC*	5.43E-07			6.90E-05	2.66E-07	0.000069809
Vancomycin	*VANRC4*	1.58E-06			7.97E-06		0.00000955
Vancomycin	*VANSC-01*				2.37E-06	6.41E-09	2.37641E-06
Vancomycin	*VANTC-01*	3.37E-06			7.07E-06		0.00001044
Vancomycin	*VANTC-02*	2.46E-07	7.33E-07	2.70E-07	3.93E-07	2.86E-08	1.6706E-06
Vancomycin	*VANTE*	1.28E-06	2.87E-07		2.39E-05		0.000025467
Vancomycin	*VANWB*	1.32E-05			1.95E-05		0.0000327
Vancomycin	*VANWG*	1.11E-06	8.33E-05	1.80E-05	3.57E-05	1.16E-05	0.00014971
Vancomycin	*VANXB*	5.99E-06	5.18E-06		1.11E-05		0.00002227
Vancomycin	*VANXD*			1.30E-06			0.0000013
Vancomycin	*VANYB*	3.86E-06	2.56E-06		9.41E-06	4.77E-07	0.000016307
Vancomycin	*VANYD-01*	3.07E-07	8.06E-08			2.72E-08	4.148E-07
other/efflux	*DFRA1*	0.001281942	0.000836378	0.000402504	0.006288249	0.000129882	0.008938955
other/efflux	*DFRA12*	2.17E-05	0.002305718	0.00069731	0.001862183	9.00E-06	0.004895911
other/efflux	*EREB*					5.19E-08	5.19E-08
other/efflux	*FOLA*	1.09E-05	9.60E-05	5.97E-06	3.48E-05	1.01E-06	0.00014868
other/efflux	*CATB8*	2.88E-06	8.41E-05	1.23E-05	2.99E-05	1.00E-06	0.00013018
MGEs	*INTI1*	0.017775432	0.020826187	0.01207137	0.019385963	0.00015387	0.070212822
MGEs	*TNPA-01*	0.000226753		0.000242879	0.000275167	6.79E-06	0.000751589

MGEs	*TNPA-02*	0.004121903	0.009035741	0.008062293	0.017884795	2.62E-05	0.039130932
MGEs	*TNPA-03*	0.003338364	0.003344863	0.00388649	0.007015696	8.88E-05	0.017674213
MGEs	*TNPA-04*	0.003610125	0.007702492	0.003122758	0.00615393	0.000110543	0.020699848
MGEs	*TNPA-05*	0.004305022	0.003814248	0.002927302	0.01049212	0.000628478	0.02216717
MGEs	*TNPA-07*	1.326403949	1.06134183	1.833699067	1.281329029	0.49215368	5.994927555

Note: different uppercase and lowercase letters on the shoulders indicate significant differences ($P < 0.05$)
